# Multimodal Communication in the Human–Cat Relationship: A Pilot Study

**DOI:** 10.3390/ani13091528

**Published:** 2023-05-03

**Authors:** Charlotte de Mouzon, Gérard Leboucher

**Affiliations:** 1 Laboratoire Ethologie Cognition Développement, Université Paris Nanterre, 92000 Nanterre, France; 2 EthoCat—Cat Behaviour Research and Consulting Institute, 33000 Bordeaux, France

**Keywords:** companion cats, *Felis catus*, social cognition, human–cat interaction, interspecific communication, multimodal communication

## Abstract

**Simple Summary:**

In a current society marked by closer relationships between humans and their pet companions, most cat owners interact with their feline partners on a daily basis. This study addresses whether, in an extraspecific interaction with humans, cats are sensitive to the communication channel used by their interlocutor. By examining three types of interactions—vocal, visual and bimodal (visual and vocal)—we found the modality of communication had a significant effect on the latency in time taken for cats to approach a human experimenter. Cats interacted significantly faster in response to visual and bimodal communication compared to vocal communication. In addition, cats displayed significantly more tail wagging when the experimenter engaged in no communication (control condition) compared to visual and bimodal communication. Taken together, our results suggest that cats display a marked preference for both visual and bimodal cues addressed by non-familiar humans compared to vocal cues only. Our findings offer further evidence for the emergence of human-compatible socio-cognitive skills in cats that favour their adaptation to a human-driven niche.

**Abstract:**

Across all species, communication implies that an emitter sends signals to a receiver, through one or more channels. Cats can integrate visual and auditory signals sent by humans and modulate their behaviour according to the valence of the emotion perceived. However, the specific patterns and channels governing cat-to-human communication are poorly understood. This study addresses whether, in an extraspecific interaction, cats are sensitive to the communication channel used by their human interlocutor. We examined three types of interactions—vocal, visual, and bimodal—by coding video clips of 12 cats living in cat cafés. In a fourth (control) condition, the human interlocutor refrained from emitting any communication signal. We found that the modality of communication had a significant effect on the latency in the time taken for cats to approach the human experimenter. Cats interacted significantly faster to visual and bimodal communication compared to the “no communication” pattern, as well as to vocal communication. In addition, communication modality had a significant effect on tail-wagging behaviour. Cats displayed significantly more tail wagging when the experimenter engaged in no communication (control condition) compared to visual and bimodal communication modes, indicating that they were less comfortable in this control condition. Cats also displayed more tail wagging in response to vocal communication compared to the bimodal communication. Overall, our data suggest that cats display a marked preference for both visual and bimodal cues addressed by non-familiar humans compared to vocal cues only. Results arising from the present study may serve as a basis for practical recommendations to navigate the codes of human–cat interactions.

## 1. Introduction

Most cat owners interact with their feline companions on a daily basis, talking, cuddling, and taking care of them. For many cats and dogs living in human environments, humans act as central partners, and companion animals often spend more time with humans than conspecifics [1,2]. Understanding the way our closest non-human companions perceive and react to their human environment is a contemporary topic which has drawn research attention in the past decade [1,3,4,5,6,7,8,9,10,11,12,13,14,15,16,17,18,19,20].

As species involved in daily interactions with humans, dogs and cats have developed “human specialized” socio-cognitive skills. These abilities have been widely investigated in dogs, showing their remarkable capacity to communicate and form relationships with humans and providing evidence for a high level of attentiveness toward humans [4,5,6,12,21,22]. By monitoring human faces, dogs obtain a flow of social information, including communicative cues and emotional and attentive states. Looking at human faces also gives them the ability to differentiate between humans, recognize familiar individuals, or even generate an internal representation of their owner’s face [23,24,25,26,27,28,29]. Regarding vocal communication, dogs are more attentive when humans talk to them using dog-directed speech, a register resembling “baby talk” [30,31,32]. Moreover, multimodal signalling in human–dog communication has been increasingly studied in recent years, with an interesting focus on contrasting command paradigms in which the vocal cues indicate an intent that mismatches visual cues [5,17,18].

Recent studies have shown that cats also have excellent socio-cognitive abilities [27]. Throughout their domestication process, cats have developed sensitivity to human communicative signals, along with human-compatible social skills that enable them to communicate with us. These skills are likely to be enhanced by life experiences. For example, cats can read human pointing gestures to locate hidden food [2] and follow the human gaze for referential information [14]. Cats also exhibit referential looking toward their owner in the presence of a potentially frightening object [33]. Additionally, they display the ability to distinguish human emotional expressions [10] and human attentional states [13,20]. Relying upon vocal cues, cats can discriminate their owner from a stranger [8,34] and recognize when speech is specifically addressed to them rather than addressed to human adults [8]. Cats can predict their owner’s face upon hearing their voice and mentally map their owner’s location relying on vocal cues, suggesting cross-modal mental representation of at least one human [19,35]. Using a cross-modal paradigm, Quaranta et al. [16] reported that, just like dogs [3] and horses [36], cats can integrate visual and auditory signals to recognize humans’ emotions, and even appear to modulate their behaviour according to the valence of the emotion perceived. 

Across all species, communication implies emitters and receivers. During a dyadic interaction, an emitter sends signals to a receiver, who decodes these signals and is able to react accordingly, sometimes turning into an emitter as well, sending so-called feedback [37]. Signals can be sent through various channels, including vocal, visual, tactile, and chemical ones. Interactions in which only one channel is involved are commonly referred to as unimodal interactions. The use of several channels is referred to as multimodal communication [38]; the use of two is known as or bimodal communication [39]. Although cat-to-cat communication has received some scientific attention, the study of cat-to-human communication is still in its early stages [40]. An example of behaviour described in cats’ intraspecific interactions is the tail-up display, an essential visual cue for cats. This behaviour, in which the tail is held up vertically without piloerection, has been shown to represent a friendly signal during a cat-to-cat approach [41,42]. Interestingly, it has been reported that cats transfer this intraspecific communicative behaviour when addressing communications to humans [40,43,44]. However, in previous studies investigating human–cat vocal communication, no tail-up displays were observed in response to human unimodal vocal signals, suggesting that unimodal vocal communication addressed by humans did not trigger visual communicative feedback in cats [8]. 

In this context, the first aim of the present study was to explore cats’ sensitivity to human cues, considering this issue through the perspective of communication modality. Knowing that cats have developed specific vocalizations for interacting with humans [40,45,46], we hypothesized that they would be keener to approach a human engaging in vocal communication compared to visual communication. Secondly, this study aimed at investigating whether, in an extraspecific interaction initiated by a human, cats adapt their communication channel to those used by their human interlocutor: in other words, whether they use the same signal modality. Based on previous observations, we hypothesized that, in the frame of their feedback to a human, cats would favour visual communicative signals in response to visual communication, and vocal communicative signals in response to vocal communication. We tested our hypotheses using three types of interactions: vocal, visual, or bimodal (visual and vocal). We also set up a control condition in which the human interlocutor refrained from emitting any communication signal.

## 2. Materials and Methods

### 2.1. Subjects

The study cohort included eighteen domestic cats (8 females and 10 males), aged 3 to 9 years (mean 5.4 ± 0.3). All were neutered cats, living in two separate cat cafés (9 cats in Bordeaux and 9 cats in Toulouse, France). These cats had been living in the cafés for at least three years and were therefore accustomed to interacting with unfamiliar humans. Two exclusion criteria were applied: cats exhibiting too many stress-related behaviours throughout habituation or test phases (e.g., head scan, escape attempt, hiding, distress vocalization [47]), and cats who never came to the experimenter (see procedure section), were excluded from the analysis. The final participants were twelve cats (6 females and 6 males) aged 3 to 7 years (mean 5.1 ± 0.3). 

### 2.2. Apparatus

**Figure 1:**
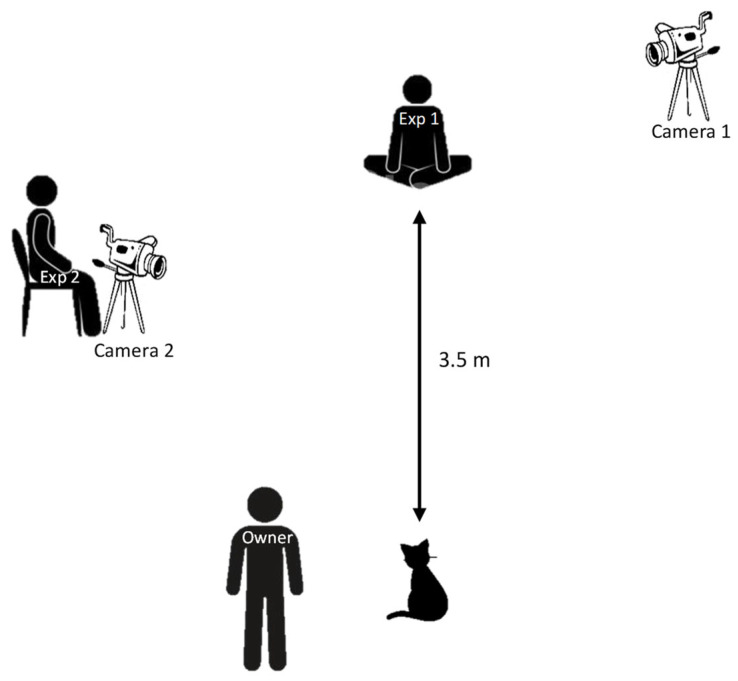
Experimental setup.

Experiments were conducted in two cat cafés, which were the everyday environment of the cat participants. Experiments were conducted in the morning, before opening the café to the public, to ensure a quiet environment. Two experimenters were present in a room separated from the rest of the café. Experimenter 1 was sitting at the end of the room and engaged in communication according to each testing condition (see procedure section). Experimenter 2 was recording videos and sat still throughout the experiments. For each test, the cat entered the room with their owner, who then stood still and was asked not to communicate with the cat. There was one owner for the cats in Bordeaux, one owner for the cats in Toulouse. The cats’ owners were also the cafés’ managers. Experiments were recorded with two video cameras (GoPro Hero CHDHB-501-RW; GoPro, Inc. San Mateo, CA, USA, News-TRONICS HD 1080P; News-TRONICS; France). Camera 1 was placed behind experimenter 1 and enabled wide-angle recording of the whole room. Camera 2 was held by experimenter 2 and focused on the cat at all times. See Figure 1 for experimental apparatus. 

### 2.3. Procedure 

#### 2.3.1. Habituation Phase 

In order for the cats to become familiar with the setup and both experimenters, a habituation phase was set up prior to the four test phases. The cat entered the room with the owner and was gently restrained for about 10 s in order to visualize the setup. Experimenter 1 was sitting at a distance of 3.5 m and was holding a treat, in order to favour a positive association. Once released, the cat could explore the room freely. Experimenter 1 also left her hand within the cat’s reach so that they could smell it or rub against it if they wished to. The habituation phase lasted until the cat came within a distance of 10 cm from experimenter 1 and for a maximum duration of 5 min. If the cat came to experimenter 1, they were verbally complimented and rewarded with a treat. If the cat did not approach experimenter 1 within 5 min, experimenter 1 gently escorted them to the door, gave them a treat and complimented them to encourage positive reinforcement, and opened the door to let them out of the room. 

#### 2.3.2. Testing Conditions 

All cats went through four testing conditions. The order of the testing conditions was randomized beforehand so it would be counterbalanced across cats. In all four testing conditions, the cat entered the room with the owner and was gently restrained for about 10 s in order to visualize the setup. Experimenter 1 sat at a distance of about 3.5 m and engaged in visual, vocal, or bimodal communication. As a control test, we also set a modality in which the experimenter engaged in no communication. Throughout testing conditions, experimenter 1 hid treats in her pocket, but did not offer the treat unless the cat came within a distance of 10 cm. If the cat came to the experimenter, they were rewarded with a treat. If the cat did not approach experimenter 1 within 75 s, they were allowed out of the room and received a treat at the door. The testing conditions were:

(a) No communication engaged (control situation): experimenter 1 did not look at or speak to the subject. She sat still and did not offer her hand.

(b) Visual communication: experimenter 1 silently offered her hand to the cat and alternated gaze directed at the cat with gaze directed at the floor. As has recently been suggested, narrowing the eyes may function as a form of positive communication between cats and humans [1]; therefore experimenter 1 engaged in slow blink sequences. 

(c) Vocal communication: experimenter 1 alternated calling the cat by their name and making cat-specific calling noises (a sort of “pff pff” sound, widely used by French humans for calling cats), but did not offer any visual interaction, for example, she did not offer her hand and she looked upwards to avoid eye contact. 

(d) Bimodal (visual and vocal) communication: experimenter 1 offered her hand to the cat and cycled between directing her gaze at the cat, slow blinks, and directing her gaze at the ground, while also calling the cat by their name and making cat-specific calling noises. 

### 2.4. Behavioural Analysis

For each testing condition, clips of the recorded videos of the cats’ responses were made using VideoPad Video Editor 7.21. A total of 48 video clips were generated, that is, one video for each testing condition for the twelve cats that went through the whole study. Videos were observed and coded using BORIS v. 7.7.3. (Behavioral Observation Research Interactive Software [48]). The ethogram was designed according to previous research [49] and a total of 11 behaviours were considered (see Table 1). As detailed in Table 1, the ethogram included both visual and vocal communicative signals, as well as behaviours indicative of the cats’ emotional state or sensitivity to the signals sent by the experimenter. All events in the ethogram were coded as “state events”, allowing exportation of the total duration for each behaviour expressed by cats under the different testing conditions. The latency of approach was assessed as the time elapsed between the owner releasing the cat and the point in time when the cat came within 10 cm of the experimenter. If a cat never came to the experimenter or expressed a desire to leave the room before the end of the test, they were assigned the maximum latency of 75 s. As not all cats’ behaviours were coded for the same duration (because, for example, cats who came in contact with experimenter 1 very quickly had their behaviours coded for a very short time), the duration of behaviours was adjusted to observation duration as follows: adjusted behaviour = (behaviour duration/observation duration) × 75 (i.e., maximum latency). This adjustment allowed comparison behaviours expressed by cats under the different testing conditions. 

### 2.5. Statistical Analysis

Statistical analysis was performed using jamovi^®^ 2.2 Computer Software [50]. The Friedman rank sum test was used to compare cats’ responses to the different testing conditions. For post-hoc comparison, responses were compared pairwise and *p*-values were calculated using Conover’s test (post-hoc Friedman–Conover test [51]). The *p*-values were adjusted using the Bonferroni method to account for multiple comparisons. Post-hoc comparisons were performed using the R package pmcmr under jamovi Rj Editor [52,53]. Two-tailed tests were used throughout; the significance level was set at alpha = 0.05.

## 3. Result

### 3.1. Latency of Approach 

**Figure 2:**
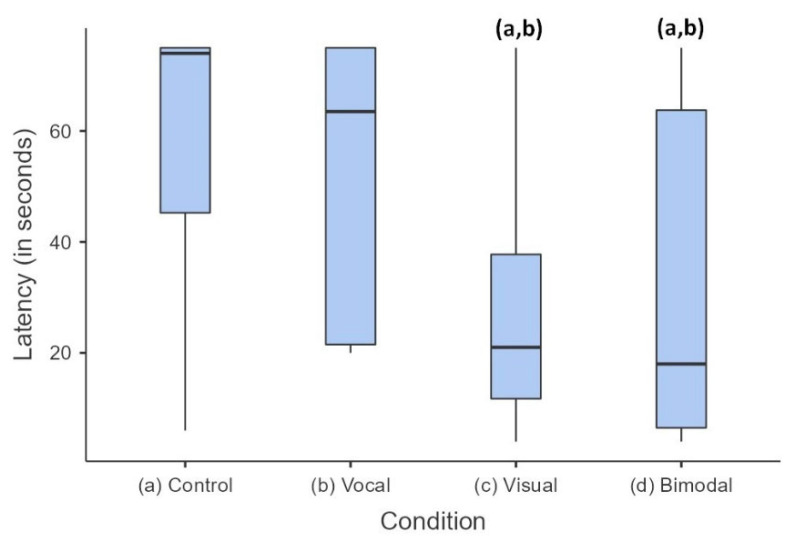
Latency in time taken for cats to approach the experimenter according to each testing condition. Median, lower and upper quartiles of the data are given; error bars represent the 10th and 90th percentiles; *n* = 12. The letters in brackets (a,b) indicate significant differences: visual (c) and bimodal (d) are different from “no communication” (a) and from vocal (b), (*p* < 0.05).

Cats’ reactions to the four types of signals i.e., vocal only, visual only, bimodal (visual + vocal) and no communication (control) were recorded. The modality of communication was observed to have a significant effect on the latency of approach (Friedman χ^2^(3,11) = 10.77, *p* = 0.013, Figure 2). Post-hoc analysis revealed that cats came significantly faster in response to visual (29 ± 6.9 s) and bimodal (32 ± 7.0 s) communication, compared to “no communication” (57.5 ± 7.6 s, *p* = 0.029 and 0.001, respectively) and vocal (51.8 ± 7.3 s, *p* = 0.045 and 0.001, respectively) modes. Among the 12 cats who approached experimenter 1 at least once throughout the trials, 6 approached in the control condition, 7 in the vocal condition, 10 in the visual condition and 9 in the bimodal condition. A detailed summary can be found in supplemental Table S1.

### 3.2. Other Behavioural Responses

**Figure 3:**
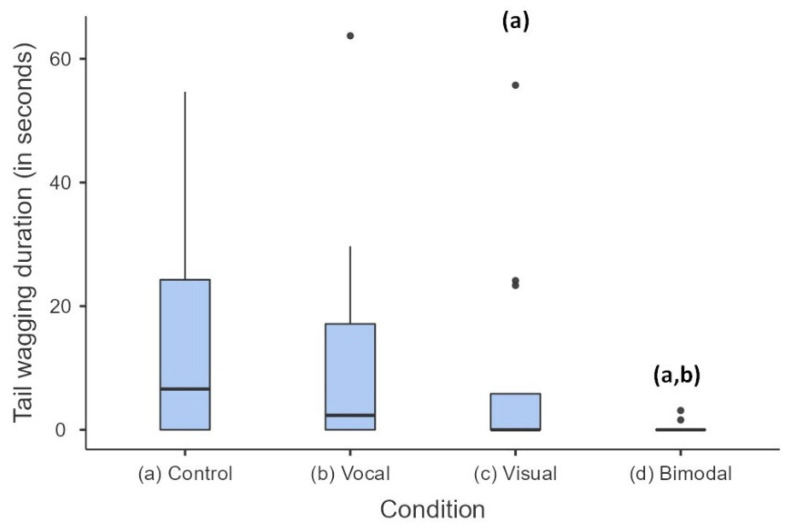
Behaviour duration according to each testing condition for cats displaying tail wagging. Behaviour durations were adjusted to observation durations. Median, lower and upper quartiles of the data are given; error bars represent the 10th and 90th percentiles; dots represent outliers; *n* = 12. The letters in brackets indicate significant differences: visual and bimodal are different from (a) “no communication”, bimodal is different from (b) vocal, (*p* < 0.05).

The cats’ behavioural responses to a human partner engaging in communication with them were analysed. Again, four modalities were examined, namely, vocal, visual, bimodal, and no communication. Tail wagging was the only behaviour significantly affected by the modality of the signal (Friedman χ^2^(3,11) = 8.812, *p* = 0.032, Figure 3). Post-hoc analysis revealed that the cats displayed significantly more tail wagging when the experimenter engaged in no communication (13.98 ± 5.21 s) compared to visual (8.6 ± 5.03 s, *p* = 0.042) and bimodal (0.39 ± 0.28 s, *p* < 0.001) communication. Cats also displayed significantly more tail wagging in vocal (11.61 ± 5.51 s) compared to bimodal communication conditions (*p* = 0.002). 

Several other of the cats’ behaviours involving vocal and visual communication were analysed: vocalizing, blinking, looking away, sniffing, grooming, locomotion, ear moving, looking at the experimenter, looking at the owner and tail up. The modality of communication used by the experimenter was found to have no significant effect on any of these behaviours (Table 2).

There were no significant differences between the two groups (Bordeaux and Toulouse) with regard to both latency (Mann–Whitney, U = 8, *p* = 0.2) and tail wagging (Mann–Whitney U = 14.5, *p* = 0.79).

## 4. Discussion

The first aim of the present study was to investigate cats’ sensitivity to vocal and visual signals sent by their human interlocutors. We hypothesized that cats would be more sensitive to vocal compared to visual signals, such that they would be keener to approach a person engaging in vocal rather than visual communication. Additionally, we investigated cats’ communicative responses to a human engaging in dyadic interactions under different modalities, in the frame of the production of feedback. Based on previous observations, we hypothesized that cats would adapt their communication channel according to those used by the human experimenter. For example, we expected more vocalizations in response to vocal communication conditions compared to visual communication condition. Similarly, we expected more tail-up behaviour in response to visual communication compared to vocal communication. 

The first outcome variable was the latency of approach – the time taken for cats to approach the human experimenter. Latency of approach can be used as an indicator of how rewarding an object or an action is to an animal [54]. A short latency of approach is thought to reflect higher attraction [55]. We observed that cats came significantly faster when the human engaged in visual or bimodal communication, compared to vocal or no communication. This suggests that cats are more sensitive to visual and bimodal communication initiated by an unfamiliar human interlocutor than when the same interlocutor engages in vocal communication or no communication at all. Our findings are in agreement with studies by Mertens and Turner [56], who reported that the latency for cats to initiate contact was higher in situations where the human was neither initiating contact nor paying attention to the cat. Furthermore, our data suggest that visual communication was more attractive than vocal communication, counter to our predictions. One hypothesis to explain this finding might by the incongruence of the signal. In the vocal-only experiment, the experimenter called the cats without looking at them. Not being accustomed to this configuration, the cats might have hesitated to approach the experimenter. This is consistent with Quaranta et al.’s findings [10] that cats were more responsive to congruent than incongruent emotional communicative signals (i.e., visual and vocal), regardless of whether they were intra- or extra-specific. As distinct species with their own specific evolutionary history, cats and dogs cannot be compared at all levels [57]. Nevertheless, these findings connect with studies exploring contrasting command paradigms in dogs [5,9,17,18]. In a recent review, Scandurra et al. [18] reported that humans’ gestures seem to be more reliable cues than words for dogs. They pointed out that word-trained dogs appear to rely more on words, whereas untrained dogs rely more on gestures. Also, visual signals trigger faster responses than auditory signals, which resembles our findings in cats.

The second group of outcome variables was the behavioural responses of the cats. Our ethogram included both vocal and visual communicative signals (e.g., vocalizations, blinking, tail-up display) and behaviours indicative of the cats’ emotional state (e.g., tail wagging, grooming). The only behaviour significantly affected by the signal modality was the tail wagging. Cats displayed more tail wagging when the human experimenter did not engage communication with them, compared to the same human offering visual or bimodal communication. We also observed more tail wagging behaviour in response to vocal compared to bimodal communication. Lateral tail movements tend to occur when cats are facing a frustrating situation [58], therefore our data suggest that being in a room with an unfamiliar human ignoring them might be uncomfortable for cats, if not frustrating. These findings also suggest that within the framework of human–cat communication, reliance on vocal cues only might be more frustrating for cats than the use of bimodal communication. The other behavioural responses examined were vocalizing, blinking, looking away, sniffing, grooming, locomotion, ear moving, looking at the experimenter, looking at the owner, and tail up. There were no significant differences according to the different modalities of communication engaged in by the experimenter. However, we cannot exclude the possibility that the absence of statistically significant differences may be due to the limited sample size of our cat cohort. 

Taken together, our results indicate that cats display a preference for visual and bimodal cues addressed by humans. These findings underline the outstanding adaptation of domestic cats to the human environment. Even though most species use several senses to communicate, they often specialize in one or two privileged senses. As signals must ultimately be detectable by the receiver, it is advantageous to convey the message using the mode of sense most highly developed in the receiver [42]. In case of extraspecific interactions, this implies identifying the favoured senses of the other species. In the context of human–cat interactions, cats, as receivers, appear to rely more upon visual cues when they need to evaluate a human’s intention to engage communication. However, it has been established that cats, as emitters, have specifically developed vocal communication modes for interacting with humans [40,45,46]. Moreover, when it comes to intraspecific interactions, cats tend to favour chemical and visual communication over vocal cues [42]. Consequently, vocal communication addressed to humans, carried by an ostensive meowing cue, is probably the best channel for cats to attract humans’ attention. Our findings thus bring further evidence for the emergence of human-compatible socio-cognitive skills in cats that favour their adaptation to a human-driven niche. Overall, cats’ adaptation to the human social environment employs a combination of ontogenetic and evolutionary processes that provide the basis for complex forms of interspecific communication.

Even though previous research has tended to report similar results amongst cats living in ordinary households and in cat-cafés [13,35], it is important consider the potential uniqueness of this experimental environment. There were two main advantages in testing cats in such conditions. First, our cat participants were accustomed to interacting and were comfortable with unfamiliar humans, which encouraged relaxed interaction with the experimenter. Furthermore, we were able to test all cats in a similar environment, thus reducing potential bias due to variability in test conditions that would have been caused by testing cats in many different households. Therefore, our results bring new insights to the general understanding of human–cat communication. Nevertheless, when investigating human–cat communication, the impact of familiarity with the human experimenter needs to be taken into account. It has been reported that cats respond differently to familiar and unfamiliar humans [8,10,19,20,34,59]. In the present study, with communication elicited by an unfamiliar human, cats seemed minimally receptive to vocal communication compared to visual and bimodal communication. We postulate that in a human–cat dyad driven by ontogenetic mechanisms cats might put more emphasis on vocal cues, whereas visual cues might come to dominate when it comes to communication with an unfamiliar human. This is similar to findings in trained dogs, with reports that visual signals are less dependent upon familiarity with the signal giver, whereas vocal signals are less effective when given by an unfamiliar person [15,16]. In a context of growing evidence for a specific attachment bond between companion cats and their owners [60,61,62,63,64], our results underline, once again, the special relationship that develops between a cat and their human, illustrated, inter alia, by the use of a particular communication into human–cat dyads. Further research may consider replicating such experiments in the frame of owner–cat interactions, jumping into this particular extraspecific relationship.

## 5. Conclusions

A better understanding of cats’ socio-cognitive abilities and human–cat communication is essential for improving the quality of human–cat relationships, as well as promoting cat welfare. Results arising from the present study may serve as a basis for practical recommendations to navigate the codes of human–cat interactions. People should be encouraged to use appropriate visual communicative cues when engaging interactions with cats, especially with unknown individuals. 

## Figures and Tables

**Table 1:** Ethogram of considered behaviours for quantification with BORIS software.

Behaviour	Description	Information
Vocalizing	Cat is producing sounds originating from the throat and mouth	Vocal signal, emotional state
Blinking	Cat blinking (eyes narrowing)	Visual signal, emotional state
Looking away	Cat is avoiding eye contact by looking elsewhere	Visual signal, emotional state
Sniffing	Cat is smelling the ground or any object	Visual signal, emotional state
Grooming	Cat is quickly licking breastplate, flank or genitals	Emotional state
Locomotion	Cat is moving around the room (more than one-step displacements)	Exploratory behaviour
Ear(s) moving	Ear movement in any direction	Sensitivity to signal
Experimenter	Cat is looking towards experimenter 1	Sensitivity to signal
Owner	Cat is looking towards owner	Emotional state
Tail wagging	Tail is slowly moving from side to side, in a continuous lateral movement	Emotional state
Tail up	Tail is held in an upright position	Visual signal, emotional state

**Table 2:** Repeated measure ANOVAs (Friedman rank-sum test) for all behavioural responses. Figures in bold highlight significant results.

Behaviour	χ^2^	df	*p*
Vocalizing	0.931	3	0.818
Blinking	6	3	0.112
Looking away	4.02	3	0.260
Sniffing	1.91	3	0.591
Grooming	5.00	3	0.172
Locomotion	4.16	3	0.245
Ear moving	4.79	3	0.188
Experimenter	5.12	3	0.163
Owner	4.23	3	0.237
Tail wagging	8.81	3	0.032
Tail up	1.05	3	0.788
Latency for approaching	10.8	3	0.013

## Data Availability

Data are available from the corresponding author on request.

## Supplementary Materials

The following supporting information can be downloaded at: https://www.mdpi.com/article/10.3390/ani13091528/s1, Table S1: Detail of cats approaching experimenter according to the testing conditions.

## References

[1] Humphrey T., Proops L., Forman J., Spooner R., McComb K. (2020). The Role of Cat Eye Narrowing Movements in Cat–Human Communication. Sci. Rep..

[2] Miklósi Á., Pongrácz P., Lakatos G., Topál J., Csányi V. (2005). A Comparative Study of the Use of Visual Communicative Signals in Interactions Between Dogs (*Canis familiaris*) and Humans and Cats (*Felis catus*) and Humans. J. Comp. Psychol..

[3] Albuquerque N., Guo K., Wilkinson A., Savalli C., Otta E., Mills D. (2016). Dogs Recognize Dog and Human Emotions. Biol. Lett..

[4] Aria M., Alterisio A., Scandurra A., Pinelli C., D’Aniello B. (2021). The Scholar’s Best Friend: Research Trends in Dog Cognitive and Behavioral Studies. Anim. Cogn..

[5] Cannas S., Mattiello S., Battini M., Ingraffia S.I., Cadoni D., Palestrini C. (2020). Evaluation of Maine Coon cat behavior during three different management situations. J. Vet. Behav..

[6] D’Aniello B., Scandurra A., Alterisio A., Valsecchi P., Prato-Previde E. (2016). The importance of gestural communication: A study of human–dog communication using incongruent information. Anim. Cogn..

[7] D’Aniello B., Fierro B., Scandurra A., Pinelli C., Aria M., Semin G.R. (2021). Sex Differences in the Behavioral Responses of Dogs Exposed to Human Chemosignals of Fear and Happiness. Anim. Cogn..

[8] De Mouzon C., Gonthier M., Leboucher G. (2023). Discrimination of Cat-Directed Speech from Human-Directed Speech in a Population of Indoor Companion Cats (*Felis catus*). Anim. Cogn..

[9] Ford G., Guo K., Mills D. (2019). Human facial expression affects a dog’s response to conflicting directional gestural cues. Behav. Proc..

[10] Galvan M., Vonk J. (2016). Man’s Other Best Friend: Domestic Cats (*F. silvestris catus*) and Their Discrimination of Human Emotion Cues. Anim. Cogn..

[11] Gergely A., Petró E., Oláh K., Topál J. (2019). Auditory–Visual Matching of Conspecifics and Non-Conspecifics by Dogs and Human Infants. Animals.

[12] Huber L., Racca A., Scaf B., Virányi Z., Range F. (2013). Discrimination of Familiar Human Faces in Dogs (*Canis familiaris*). Learn. Motiv..

[13] Ito Y., Watanabe A., Takagi S., Arahori M., Saito A. (2016). Cats beg for food from the human who looks at and calls to them: Ability to understand humans’ attentional states. Psychologia.

[14] Pongrácz P., Szapu J.S., Faragó T. (2019). Cats (*Felis silvestris catus)* Read Human Gaze for Referential Information. Intelligence.

[15] Prato-Previde E., Cannas S., Palestrini C., Ingraffia S., Battini M., Ludovico L.A., Ntalampiras S., Presti G., Mattiello S. (2020). What’s in a Meow? A Study on Human Classification and Interpretation of Domestic Cat Vocalizations. Animals.

[16] Quaranta A., d’Ingeo S., Amoruso R., Siniscalchi M. (2020). Emotion Recognition in Cats. Animals.

[17] Scandurra A., Alterisio A., Marinelli L., Mongillo P., Semin G.R., D’Aniello B. (2017). Effectiveness of verbal and gestural signals and familiarity with signal-senders on the performance of working dogs. Appl. Anim. Behav. Sci..

[18] Scandurra A., Pinelli C., Fierro B., Di Cosmo A., D’Aniello B. (2020). Multimodal signaling in the visuo-acoustic mismatch paradigm: Similarities between dogs and children in the communicative approach. Anim. Cogn..

[19] Takagi S., Arahori M., Chijiiwa H., Saito A., Kuroshima H., Fujita K. (2019). Cats Match Voice and Face: Cross-Modal Representation of Humans in Cats (*Felis catus*). Anim. Cogn..

[20] Vitale K.R., Udell M.A.R. (2019). The Quality of Being Sociable: The Influence of Human Attentional State, Population, and Human Familiarity on Domestic Cat Sociability. Behav. Proc..

[21] Gácsi M., McGreevy P., Kara E., Miklósi Á. (2009). Effects of Selection for Cooperation and Attention in Dogs. Behav. Brain Funct..

[22] Hare B., Tomasello M. (2005). Human-like Social Skills in Dogs?. Trends Cogn. Sci..

[23] Adachi I., Kuwahata H., Fujita K. (2007). Dogs Recall Their Owner’s Face upon Hearing the Owner’s Voice. Anim. Cogn..

[24] Call J., Bräuer J., Kaminski J., Tomasello M. (2003). Domestic Dogs (*Canis familiaris)* Are Sensitive to the Attentional State of Humans. J. Comp. Psychol..

[25] Gácsi M., Miklósi Á., Varga O., Topál J., Csányi V. (2004). Are Readers of Our Face Readers of Our Minds? Dogs (*Canis familiaris*) Show Situation-Dependent Recognition of Human’s Attention. Anim. Cogn..

[26] Miklösi Á., Polgárdi R., Topál J., Csányi V. (1998). Use of Experimenter-given Cues in Dogs. Anim. Cogn..

[27] Racca A., Amadei E., Ligout S., Guo K., Meints K., Mills D. (2010). Discrimination of Human and Dog Faces and Inversion Responses in Domestic Dogs (*Canis familiaris*). Anim. Cogn..

[28] Schwab C., Huber L. (2006). Obey or Not Obey? Dogs (*Canis familiaris*) Behave Differently in Response to Attentional States of Their Owners. J. Comp. Psychol..

[29] Soproni K., Miklósi A., Topál J., Csányi V. (2001). Comprehension of Human Communicative Signs in Pet Dogs (*Canis familiaris*). J. Comp. Psychol..

[30] Ben-Aderet T., Gallego-Abenza M., Reby D., Mathevon N. (2017). Dog-Directed Speech: Why Do We Use It and Do Dogs Pay Attention to It?. Proc. R. Soc. B.

[31] Benjamin A., Slocombe K. (2018). ‘Who’s a Good Boy?!’ Dogs Prefer Naturalistic Dog-Directed Speech. Anim. Cogn..

[32] Jeannin S., Gilbert C., Amy M., Leboucher G. (2017). Pet-Directed Speech Draws Adult Dogs’ Attention More Efficiently than Adult-Directed Speech. Sci. Rep..

[33] Merola I., Lazzaroni M., Marshall-Pescini S., Prato-Previde E. (2015). Social Referencing and Cat–Human Communication. Anim. Cogn..

[34] Saito A., Shinozuka K. (2013). Vocal Recognition of Owners by Domestic Cats (*Felis catus*). Anim. Cogn..

[35] Takagi S., Chijiiwa H., Arahori M., Saito A., Fujita K., Kuroshima H. (2021). Socio-Spatial Cognition in Cats: Mentally Mapping Owner’s Location from Voice. PLoS ONE.

[36] Trösch M., Cuzol F., Parias C., Calandreau L., Nowak R., Lansade L. (2019). Horses Categorize Human Emotions Cross-Modally Based on Facial Expression and Non-Verbal Vocalizations. Animals.

[37] Beauchaud M., Darmaillacq A.S., Lévy F. (2020). La Communication Animale. Ethologie Animale. Une Approche Biologique du Comportement.

[38] Partan S., Marler P. (1999). Communication Goes Multimodal. Science.

[39] Baraud I., Deputte B.L., Pierre J.-S., Blois-Heulin C., Magnusson M.S., Burgoon J.K., Casarrubea M. (2016). Informative Value of Vocalizations during Multimodal Interactions in Red-Capped Mangabeys. Discovering Hidden Temporal Patterns in Behavior and Interaction.

[40] Turner D.C. (2021). The Mechanics of Social Interactions Between Cats and Their Owners. Front. Vet. Sci..

[41] Cafazzo S., Natoli E. (2009). The Social Function of Tail up in the Domestic Cat (*Felis silvestris catus*). Behav. Proc..

[42] Cameron-Beaumont C. (1997). Visual and Tactile Communication in the Domestic Cat (*Felis silvestris catus*) and Undomesticated Small Felids. Ph.D. Thesis.

[43] Crowell-Davis S., Rochlitz I. (2007). Cat Behaviour: Social Organization, Communication and Development. The Welfare of Cats.

[44] Deputte B.L., Jumelet E., Gilbert C., Titeux E. (2021). Heads and Tails: An Analysis of Visual Signals in Cats, *Felis catus*. Animals.

[45] Bradshaw J., Cameron-Beaumont C., Turner D., Bateson P. (2000). The Signalling Repertoire of the Domestic Cat and Its Undomesticated Relatives. The Domestic Cat: The Biology of Its Behaviour, 2nd ed.

[46] Yeon S.C., Kim Y.K., Park S.J., Lee S.S., Lee S.Y., Suh E.H., Houpt K.A., Chang H.H., Lee H.C., Yang B.G. (2011). Differences between Vocalization Evoked by Social Stimuli in Feral Cats and House Cats. Behav. Proc..

[47] Nibblett B.M., Ketzis J.K., Grigg E.K. (2015). Comparison of Stress Exhibited by Cats Examined in a Clinic versus a Home Setting. Appl. Anim. Behav. Sci..

[48] Friard O., Gamba M. (2016). BORIS: A Free, Versatile Open-source Event-logging Software for Video/Audio Coding and Live Observations. Methods Ecol. Evol..

[49] Stanton L.A., Sullivan M.S., Fazio J.M. (2015). A Standardized Ethogram for the Felidae: A Tool for Behavioral Researchers. Appl. Anim. Behav. Sci..

[50] (2021). The Jamovi Project, Jamovi Version 2.2. https://www.jamovi.org.

[51] Pereira D.G., Afonso A., Melo Medeiros F. (2015). Overview of Friedman’s test and post-hoc analysis. Commun. Stat. -Simul. Comput..

[52] R Core Team (2021). R: A Language and Environment for Statistical Computing.

[53] Pohlert T. (2018). PMCMR: Calculate Pairwise Multiple Comparisons of Mean Rank Sums. [R Package]. https://cran.r-project.org/package=PMCMR.

[54] Ratuski A.S., Makowska I.J., Dvorack K.R., Weary D.M. (2021). Using Approach Latency and Anticipatory Behaviour to Assess Whether Voluntary Playpen Access Is Rewarding to Laboratory Mice. Sci. Rep..

[55] Bensoussan S., Tigeot R., Lemasson A., Meunier-Salaün M.-C., Tallet C. (2019). Domestic Piglets (*Sus scrofa domestica*) Are Attentive to Human Voice and Able to Discriminate Some Prosodic Features. Appl. Anim. Behav. Sci..

[56] Mertens C., Turner D.C. (1988). Experimental Analysis of Human-Cat Interactions During First Encounters. Anthrozoös.

[57] Uccheddu S., Miklósi Á., Gintner S., Gácsi M. (2022). Comparing Pears to Apples: Unlike Dogs, Cats Need Habituation before Lab Tests. Animals.

[58] Kiley-Worthington M. (1976). The Tail Movements of Ungulates, Canids and Felids With Particular Reference To Their Causation and Function as Displays. Behaviour.

[59] Ellis S.L.H., Swindell V., Burman O.H.P. (2015). Human Classification of Context-Related Vocalizations Emitted by Familiar And Unfamiliar Domestic Cats: An Exploratory Study. Anthrozoös.

[60] Bouma E.M.C., Reijgwart M.L., Dijkstra A. (2021). Family Member, Best Friend, Child or ‘Just’ a Pet, Owners’ Relationship Perceptions and Consequences for Their Cats. IJERPH.

[61] Eriksson M., Keeling L.J., Rehn T. (2017). Cats and Owners Interact More with Each Other after a Longer Duration of Separation. PLoS ONE.

[62] Finka L.R., Ward J., Farnworth M.J., Mills D.S. (2019). Owner Personality and the Wellbeing of Their Cats Share Parallels with the Parent-Child Relationship. PLoS ONE.

[63] Vitale K.R., Behnke A.C., Udell M.A.R. (2019). Attachment Bonds between Domestic Cats and Humans. Curr. Biol..

[64] Vitale Shreve K.R., Mehrkam L.R., Udell M.A.R. (2017). Social Interaction, Food, Scent or Toys? A Formal Assessment of Domestic Pet and Shelter Cat (*Felis silvestris catus*) Preferences. Behav. Proc..

